# A workflow to investigate the impacts of weathered multi-walled carbon nanotubes to the mud snail *Lymnaea stagnalis*

**DOI:** 10.1007/s11356-021-17691-0

**Published:** 2021-12-02

**Authors:** Katrin Weise, Thomas Kurth, Irina Politowski, Carola Winkelmann, Andreas Schäffer, Susanne Kretschmar, Thomas Ulrich Berendonk, Dirk Jungmann

**Affiliations:** 1 Institute for Hydrobiology, Faculty of Environmental Sciences, Technische Universität Dresden, Zellescher Weg 40, 01217 Dresden, Germany; 2 Technology Platform, Center for Molecular and Cellular Bioengineering (CMCB), Technische Universität Dresden, Fetscherstraße 105, 01307 Dresden, Germany; 3 Institute for Environmental Research, RWTH Aachen University, Worringerweg 1, 52074 Aachen, Germany; 4 Institute for Integrated Natural Sciences, University of Koblenz-Landau, Universitätsstraße 1, 56070 Koblenz, Germany

**Keywords:** *L. stagnalis*, Benthic biofilm, CNTs, wMWCNTs, Enrichment, Physiological state, Histology

## Abstract

Although the development and application of nanomaterials is a growing industry, little data is available on the ecotoxicological effects on aquatic organisms. Therefore, we set up a workflow to address the potential uptake of weathered multi-walled carbon nanotubes (wMWCNTs) by a model organism, the pulmonary mud snail *Lymnaea stagnalis* (*L. stagnalis*), which plays an important role in the food web. It represents a suitable organism for this approach because as a grazer it potentially ingests large amounts of sedimented wMWCNTs. As food source for *L. stagnalis*, benthic biofilm was investigated by the use of a transmission electron microscope (TEM) and a scanning electron microscope (SEM) after exposure with wMWCNTs. In addition, isotopic labeling was applied with ^14^C-wMWCNTs (0.1 mg/L) to quantify fate, behavior, and enrichment of ^14^C-wMWCNTs in benthic biofilm and in *L. stagnalis*. Enrichment in benthic biofilm amounted to 529.0 µg wMWCNTs/g dry weight and in *L. stagnalis* to 79.6 µg wMWCNTs/g dry weight. A bioconcentration factor (*BCF*) for *L. stagnalis* was calculated (3500 L/kg). We demonstrate the accumulation of wMWCNTs (10 mg/L) in the digestive tract of *L. stagnalis* in an effect study. Moreover, the physiological markers glycogen and triglycerides as indicators for the physiological state, as well as the RNA/DNA ratio as growth indicator, were examined. No significant differences between exposed and control animals were analyzed for glycogen and triglycerides after 24 days of exposure, but a decreasing trend is recognizable for triglycerides. In contrast, the significant reduction in the RNA/DNA ratio of *L. stagnalis* indicated an inhibition of growth with a following recovery after depuration. The described workflow enables a comprehensive determination of the fate and the behavior of wMWCNTs specifically and in general all kinds of CNTs in the aquatic environment and therefore contributes to a holistic risk assessment of wMWCNTs.

## Introduction

Nanotechnology is an increasing sector in the industry with a wide range of applications such as drug delivery, healthcare products, or textiles (Baun et al. 2008; Bianco et al. 2005; Schwirn and Völker 2016). One product line are CNTs which can be divided into three main groups: single-, double-, and multi-walled carbon nanotubes (SWCNTs, DWCNTs, and MWCNTs, Petersen et al. 2011). CNTs, especially MWCNTs, are described to be the star in the nanomaterials industry (Sebastian et al. 2013). These groups are found in wastewater at concentrations between 3.69 and 32.66 ng/L, whereas a range of 0.001 and 0.8 ng/L has been detected in surface waters (Maurer-Jones et al. 2013), but environmental concentrations of nanomaterials in general are widely unknown (Lawrence et al. 2016). Due to agglomeration and sedimentation, over time, CNTs deposit in water and therefore cause higher concentrations in sludge and sediment (Chen et al. 2010; Schierz et al. 2014; Glomstad et al. 2018; Gottschalk et al. 2009; Sun et al. 2014; Politowski et al. 2021a). For this, reported CNT concentrations in sediment compartments range between 1 µg/kg and 1 mg/kg (Selck et al. 2016).

CNTs are attributed to their special sorption characteristics (Mueller and Nowack 2008; Petersen et al. 2016). Depending on pH, temperature, or redox processes, CNTs are able to adsorb hydrophobic environmental chemicals, which might promote a deviant accumulation behavior in the environment, for both the sorbent and the sorbate. MWCNTs directly reach the aquatic environment via, e.g., industrial production, whereas the indirect introduction occurs via sewage and landfill leachates (Petersen et al. 2011). The structure of MWCNTs in the aquatic environment is affected by natural processes, like oxidative photochemical or bacterial degradation (Klaine et al. 2008), accompanied with aging effects due to irradiation. Hence, it is crucial to understand the fate and behavior of MWCNTs in the environment. For this, combined experiments for hazard analysis of these interactions and accumulation properties of MWCNTs must be established.

Organisms in the aquatic environment are basically exposed via two pathways, uptake via water (bioconcentration) or food (biomagnification). Taking fate and behavior of MWCNTs into account for hazard identification, an experimental design is needed which considers relevant organisms and food web interactions (Mortimer et al. 2016; Cano et al. 2018). In lakes and rivers, benthic biofilms with their microbial community (Characklis et al. 1982) play a considerable role in the food web. Such biofilms consist of taxa from many different phyla including bacteria, algae (e.g., diatoms), fungi, ciliates, cyanobacteria, rotifers, and nematodes (Kohušová et al. 2011). For single species, numerous publications on the effects of CNTs on these organisms exist, but effects on interactions or the transfer of CNTs within the food web have been less investigated (Politowski et al. 2021a). Organisms depending on biofilm as food source occur among the functional feeding group of grazers. Different macroinvertebrates appertain to this group, especially snails and mayflies. By combining biofilm and grazers in an experimental approach, the impact of MWCNTs on the food web transfer of MWNCTs as well as their distribution, behavior, and accumulation can be analyzed for hazard assessment. In order to consider environmental aging processes of nanomaterials, in our study, we focused on wMWCNTs.

The first step in our study was to investigate possible structural differences between wMWCNTs and pristine MWCNTs (MWCNTs) and additionally the enrichment of wMWCNTs in benthic biofilm. The impact of MWCNTs on primary producers, stress phenomena, and changes in the composition of algal cells was investigated in various publications (Long et al. 2012; Rhiem et al. 2015; Schwab et al. 2011; Wang et al. 2019; Politowski et al. 2021b). However, higher-tier approaches have been neglected, and the available approaches are not necessarily applicable to investigate food web interactions. For primary consumers, such as the water flea *Daphnia magna* (*D. magna*), it has been shown that CNTs can accumulate in their body and that depuration is delayed in the absence of food (Petersen et al. 2009; Tervonen et al. 2010; Politowski et al. 2021b). Guo et al. (2013) investigated the depuration and the uptake of radiolabeled graphene by *D. magna* and described the release of significant fractions (~ 90%) of accumulated graphene after the addition of algae and humic acid during a depuration period. It has also been shown that CNTs from food matrix, or sediment, are accumulated in aquatic invertebrates (Li et al. 2010; Wang et al. 2014; Petersen et al. 2008; Parks et al. 2013). For fish, quick uptake, accumulation, and depuration of CNTs in the intestine have been shown, while only a few fragments of CNTs reached the blood system and muscle tissue (Maes et al. 2014). Therefore, a more comprehensive assessment of environmental risks for animals are highly relevant due to their ability to enrich MWCNTs contingent on the key role in the food web.

Thus, the second step was to include a primary consumer into the test approach that feeds directly on benthic biofilms. The freshwater snail *Lymnaea stagnalis* (Gastropoda: Lymnaeidae) was selected as test organism, due to its wide distribution in standing and flowing waters of the northern hemisphere and its crucial role in the aquatic and rural food web (Nyström and Pérez 1998; Orr et al. 2007). In addition, invertebrates are described as more sensitive, unlike vertebrates (Jackson et al. 2013). Daoud et al. (2021) investigated the oxidative stress and genotoxicity parameters of the snail *Lymnaea luteola* (*L. luteola*) in vivo and described snails as a useful tool to screen toxic potentials of environmental contaminations. The organism *L. stagnalis* ingests all kinds of contaminated material from biofilms, and it is vulnerable to dissolved or particle-adsorbed pollutants (Lance et al. 2010). Because of their high sensitivity towards toxins, over the past two decades, this species has been proven particularly useful to study the toxicological effects of aquatic contaminants (Amorim et al. 2019). Thus, it is an ideal model for our study.

Consequently, we set up a workflow to investigate different effects of wMWCNTs to *L. stagnalis* and to address whether wMWCNTs are taken up by the snail from benthic biofilm. For this, wMWCNTs were investigated in a quantification study for benthic biofilm and for *L. stagnalis* each via bioconcentration. Therefore, we used custom synthesized weathered radioactively labeled wMWCNTs (^14^C-wMWCNT). To investigate their potential to induce a reduction of physiological functions, we quantified the physiological markers glycogen and triglycerides (TGs) as the main substrates to store energy in animals and therefore prerequisites for future growth, as well as resilience or reproduction in insects, crustaceans (Koop et al. 2008), and snails (Nicolai et al. 2012). The reasoning behind the physiological markers is that a low amount of stored energy would indicate high energetic costs of the exposure either due to higher expenditure or due to lower uptake (Koop et al. 2011). While TGs provide more adenosine triphosphate (ATP) per mass, the metabolism of glycogen is independent on the presence of oxygen (Koop et al. 2011). Because the mode of the physiological answer is not yet clear for *L. stagnalis*, both markers were analyzed. We also determined the RNA/DNA ratio as an indicator of the current growth intensity, referring to the actual investment and anabolic activity of organisms (Koop et al. 2011).

Furthermore, we want to know the fate of accumulated wMWCNTs in *L. stagnalis* and their distribution in the organism because after ingestion and passage through the esophagus, food reaches the strongly twisted intestine, which is covered by the digestive gland and where the intracellular digestion takes place. We monitored the accumulated wMWCNTs in the lumen of the intestinal tract by histological analyses and its potential association with gut epithelial cells using TEM.

## Materials and methods

### Microcosm and established benthic biofilm

**Fig. 1:**
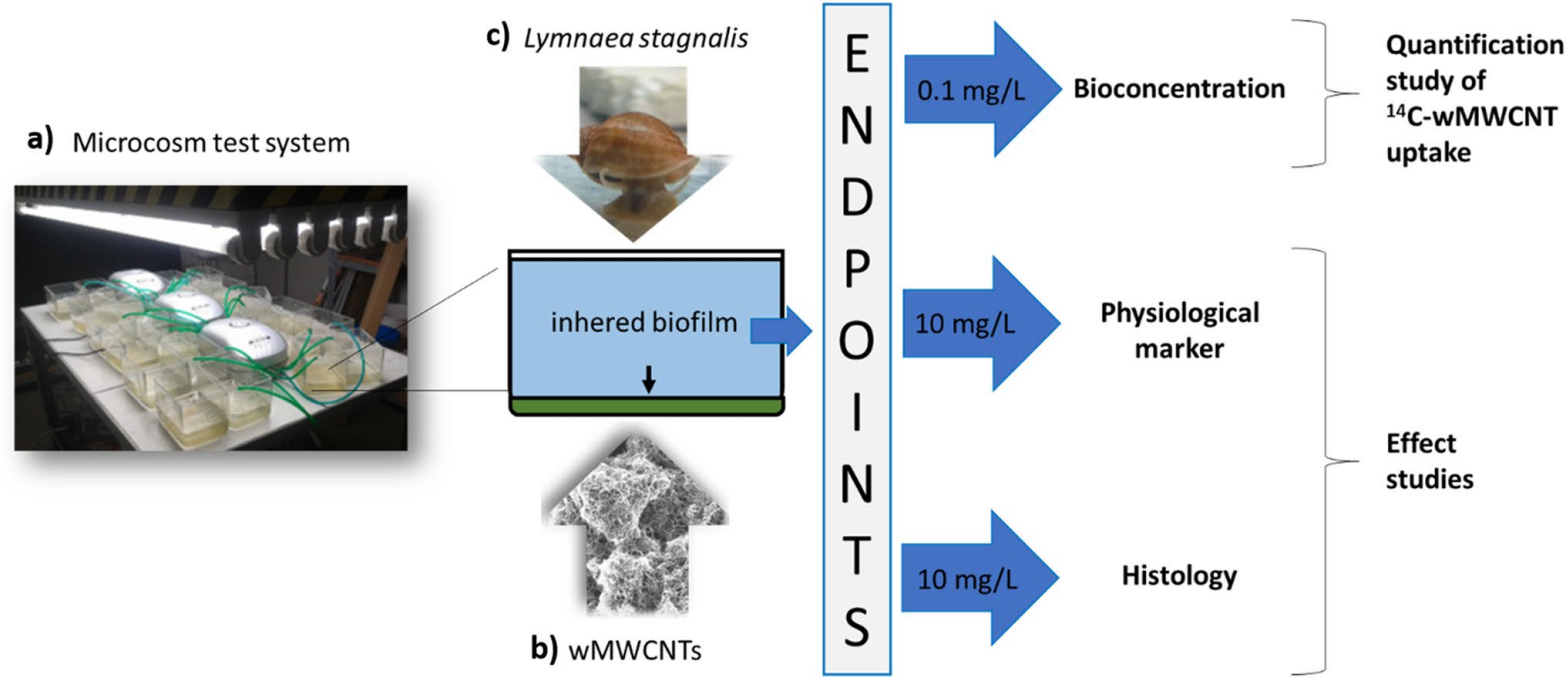
Overview of the whole workflow in the experimental setup: **a** microcosm, **b** wMWCNTs (SEM), and **c** *L. stagnalis* (alive image). Arrows indicates the different exposures of 0.1 and 10 mg/L together with the chosen methods in the quantification and effect study

One microcosm system (Fig. 1a) for both test studies (quantification and effect study) consisted of 16 glass aquaria (10 × 14.4 × 11 cm). Experiments took place in air-conditioned laboratories at a constant temperature of 20 ± 1 °C with a light/dark cycle of 12/12 h. For quantification and effect studies, the benthic biofilm was sampled in the Gauernitzbach, a second-order mountain stream of 4.6 km length and tributary of the River Elbe, which has been described in detail by Winkelmann and Koop (2007). The stream catchment is moderately affected by urban and rural impacts (Kroll et al. 2016). By scraping off stones from the stream, some biofilm was obtained. The harvested biofilm was then further treated as described by Rybicki et al. (2012). A mixed solution of biofilm suspension and Borgmann medium in a ratio of 3:1 (biofilm suspension/Borgmann medium) was applied to ensure a better adhesion and growth of the biofilm. This solution was modified for the quantification and effect study with one-eighth of the amount of CaCO_3_ compared to Borgmann (1996) due to adsorption properties (Kroll et al. 2016; Rybicki et al. 2012), in both test studies. An aliquot was added to each aquarium, where it was allowed to sediment to the bottom for 24 h prior to the test start of the quantification and effect study, respectively.

### Test substance wMWCNT

The applied test substance was identified via SEM with its typically crosslink structure (Fig. 1b). Synthesis and weathering of MWCNTs (Baytubes C 150 P, BTS, Leverkusen, Germany) were purchased from Bayer MaterialScience AG 2007 (details are depicted in Table 1). The radiolabeled ^14^C-MWCNTs were synthesized as described by Maes et al. (2014) and Rhiem et al. (2015) at the Institute for Environmental Research at RWTH Aachen in collaboration with Bayer Technology Services GmbH (BTS, Leverkusen, Germany). Afterwards, ^14^C-MWCNTs were purified using 12.5% hydrochloric acid solution to remove residual metal catalyst. Weathering of labeled and non-labeled MWCNTs was performed in a Sunset CPS + apparatus (ATLAS Material Testing Solutions) applying ultraviolet radiation for about 3 months (65 W/m^2^ = 504,440 kJ/m^2^) according to ISO 3892–2:2006. The device provided light with a wavelength range from 300 to 400 nm by means of an air-cooled xenon lamp (1500 W) with a daylight UV filter. Irradiation was performed without simulated rain or air humidity. During weathering, the internal sample table was cooled with a constant flow of cold water. The samples were shaken daily, and the position of sample bins in the testing apparatus was changed once a week in order to achieve a uniform irradiation. After weathering process, the specific radioactivity of ^14^C-labeled wMWCNTs was determined to 1.66 MBq/mg (corresponding to 99,858 dpm/µg). To characterize the wMWCNT, thermogravimetric analysis (TGA) and Fourier-transform infrared spectroscopy (FTIR) methods were used. For more details on determination of specific radioactivity and characterization of ^14^C-wMWCNTs, see Politowski et al. (2021a, b).

**Table 1:** Properties of the appointed MWCNTs (Baytubes, purity of > 95%), divided by values and units

	Value	Unit
Number of walls	3–15	–
Outer diameter distribution	5–20	nm
Inner diameter distribution	2–6	nm
Length	1–10	µm
Bulk density	140–160	kg/m^3^

### Test organism* L. stagnalis*

Living individuals of *L. stagnalis* (Fig. 1c) were obtained from the breeding station INRA (French National Institute for Agricultural Research, France) for all experiments. They were reared in Borgmann medium according to the recipe of LO-4S E + H (Borgmann 1996) and fed three to four times a week with small pieces of cucumber and salad (organic). The medium in the aquaria was renewed once a week with fresh Borgmann medium. Before starting the quantification and effect study, the mollusks were adapted 3 weeks to Borgmann medium modified with one-eighth of the amount of CaCO_3_ (Borgmann 1996; Kroll et al. 2016; Rybicki et al. 2012). All further physical parameters were applied according to the OECD guideline of reproduction tests for *L. stagnalis* (OECD 2016). For the quantification and effect studies, 160 animals with a mean shell length of 11.5 ± 2.4 mm were used, i.e., 80 individuals being taken per test study. Oxygen was introduced into the aquaria at 16 L/min via a Pasteur pipette, which was mounted on a tube and an air pump (Hailea Aco 9630) to ensure not less than 60% of oxygen during the whole experiments.

### General experimental setup

The two different test studies (Fig. 1) were accompanied by mortality investigation. The quantification study (bioconcentration) was carried out with ^14^C-wMWCNTs in benthic biofilm during 168 h and *L. stagnalis* during 72 h. The effect study was realized with unlabeled wMWCNTs over 52 days whereby an exposure time for 24 days and a following depuration about 28 day was implemented. This study was divided into physiological and histology methods accompanied with prior performed microscopy between wMWCNT and MWCNT structure, benthic biofilm, and *L. stagnalis*. Controls were examined parallel to all methods.

### Quantification study

#### Quantification of ^14^C-wMWCNT uptake in benthic biofilm

A fresh prepared stock dispersion with a concentration of 0.1 mg/L ^14^C-wMWCNTs was chosen. For this, a total amount of 1.014 mg ^14^C-wMWCNT agglomerates was weighed using a microbalance (RADWAG, DE), transferred to a flask, and filled with 101.4 mL distilled water. Afterwards, a dispersion for 10 min by an ultrasonic probe (Sonopuls HD 2070, 70 W, pulse: 0.2 s, pause: 0.8 s, Bandelin, Germany) was applied. After sonication, 1 mL of the stock dispersion was transferred into a vial, filled with LSC cocktail in a ratio of 1:1 (Perkin, Elmer, Ultima Gold XR, 6,013,119), and measured by using the Liquid Scintillation Analyzer (LSC, Hidex 600/300 SL, Finland). For each sampling point (4 h, 24 h, 120 h, and 168 h), including four replicates, 20.4 mL from the stock dispersion (described above) was transferred to 999.6 mL freshly prepared modified Borgmann medium and dispersed again as aforementioned. All samples of ^14^C-wMWCNT were measured by taking five aliquots of 1 mL using the LSC (see above), directly after sonication.

The supernatant in the aquaria of prior bonded biofilm was removed with a custom-made glass U-tube. To obtain a volume of 400 mL as abovementioned, a volume of 200 mL from the ^14^C-wMWCNT dispersion together with 200 mL fresh Borgmann medium was added to each aquarium. Thereby, the solution was carefully poured along the aquarium glass wall. Sampling was performed in four aquaria as replicates, respectively. Initially, the whole water body from each aquarium was removed as described above. A syringe attached to the glass U-tube was used to suck the water phase out of the aquarium, without whirling up the biofilm. After that, the whole water phase was transferred to a 500 mL Schott flask. Subsequently, the water phase was dispersed again for 10 min by sonication (see above). Afterwards, five aliquots of 10 g from each Schott flask were drawn from the water phase and measured again by means of LSC. The remaining biofilm was completely scraped out of the aquaria with a spatula and dried for 24 h in a petri dish at 100 °C in a drying cabinet (Memmert, DE). Afterwards the whole dried biofilm was weighed (analytical balance, Sartorius MC1 AC210S) and split into three vials (10 mL). After that, the vials were filled with LSC cocktail in a ratio of 1:1 and measured by means of LSC. Additionally, each whole aquarium was aspired, to collect all the radioactivity of the residuals. Residuals consisted of three parts. The first part was the phase, consisting of the supernatant fluid that could not be harvested with the glass U-tube before scraping out the biofilm. Second, the aquaria were wiped out with a cellulose cloth imbued with methanol (VWR, Germany) for two times, and third, the radioactivity was adsorbed to the Pasteur pipette, which was in contact with the water phase for applying oxygen. A recovery rate was calculated for the whole experimental setup to obtain the quantity of all radioactivity of each aquarium.

In addition, a risk quotient (*RQ*) for the risk assessment was calculated with the following equation (Eq. 1): $RQ=\mathrm{PEC }/\mathrm{ PNEC}$, whereby *RQ* is classified as the risk characterization ratio. This ratio is calculated by dividing the predicted or measured environmental concentration (*PEC* or *MEC*, mg/L) through the extrapolated effect concentration (predicted no effect concentration; *PNEC*, mg/L) (Mathes 1997). An uncertainty factor to extrapolate the *PNEC* from the lowest found effect value depends on existing data for MWCNTs and is described in detail by the Technical Guidance Document (European Chemical Bureau 2003).

#### Quantification of ^14^C-wMWCNT uptake in *L. stagnalis*

For the stock dispersion in the quantification study with *L. stagnalis*, a total amount of 1356 mg ^14^C-wMWCNTs was weighed for the sampling points (4 h, 24 h, 48 h, and 72 h) and dispersed in 135.6 mL distilled water. The same procedure was used as for benthic biofilm but with shorter incubation times (4 h, 24 h, 48 h, and 72 h) to prevent starvation of the animals. The samples of ^14^C-wMWCNT were measured after sonication (described above).

Additionally, to avoid snails from creeping out of the aquarium, a net was used as cover. Afterwards, five snails after adaption (see above) were transferred to each aquarium. At every sampling, all *L.* *stagnalis* from each aquarium were transferred to a petri dish with a tweezer and filled with methanol (VWR, Germany), to kill the snails. Thereafter, the whole water phase was removed as described above. The shell of each individual organism was removed from the tissue with a tweezer and placed on a petri dish. Subsequently, both (shell and tissue) were dried separately for 24 h at 100 °C in a drying cabinet (Memmert, DE) and weighed afterwards (analytical balance, Sartorius MC1 AC210S). The tissue and the shell were crushed in a glass mortar grinder separately. The dried material was transferred into LSC vials and filled up with LSC cocktail in a ratio of 1:1. In the following steps, the shells and the tissues were measured separately for each snail with LSC. Moreover, residuals like cellulose cloth imbued with methanol (VWR, Germany) and Pasteur pipette were measured (described above) by means of LSC as well. Additionally, all excrements of *L. stagnalis* were investigated. For this, all excrements from each aquarium were collected from the bottom of each aquarium, transferred into vials, filled up with LSC cocktail (ratio 1:1), and measured by means of LSC. Furthermore, at sampling point of 72 h, the net potentially contaminated with radioactivity was added to the residuals. For this, the cellulose cloth was used to absorb the radioactivity from the net. Equally, a recovery rate was also calculated for all sampling points together with the water phase, tissue, shell, excrements, and residuals. Moreover, a bioconcentration factor (*BCF*) was calculated using the following equation (Eq. 2): $BCF=\mathrm{c}(\mathrm{snail}-\mathrm{tissue})/\mathrm{c}(\mathrm{water}) [\mathrm{L}/\mathrm{kg}]$.

### Effect studies

#### TEM and SEM investigation of MWCNTs, wMWCNTs, and benthic biofilm

TEM (Libra120, Carl Zeiss Microscopy GmbH, Oberkochen, Germany) operated at 120 kV acceleration voltage and SEM (NEON40, Carl Zeiss Microscopy GmbH, Oberkochen, Germany) prosecuted at 3 kV acceleration voltage were used for a structure analyses of the stock solution of MWCNTs and wMWCNTs. For TEM of MWCNTs in water, two microliters of liquid were dropped on plasma-hydrophilized TEM grids and dried at room temperature. For SEM of MWCNTs in water, five microliters of liquid were dropped on plasma-hydrophilized 5 mm × 5 mm silicon wafers and dried at room temperature. The silicon wafers were mounted with double-sided conductive tape on SEM sample stamps. To investigate the impact of wMWCNTs on biofilm structure, an exposure approach with 0.1 mg/L wMWCNTs was performed. The biofilm was sampled from Gauernitzbach and allowed to grow on glass slides for 1 week in an analogous manner as described above added with 0.1 mg/L wMWCNTs. For SEM of the biofilm, small pieces of biofilm-coated glass slides were broken off and mounted with double-sided conductive tape on SEM sample stamps. All samples were coated with 20 nm carbon (SCD500 coater, Leica Microsystems GmbH, Germany) to reduce charging under the electron beam.

#### Visual examination of *L. stagnalis*

To get a conception how *L. stagnalis* was affected by wMWCNTs, a prior test with benthic biofilm–contaminated wMWCNTs (10 mg/L) was investigated visually. For this, *L.* *stagnalis* was grazed over 7 days on it and analyzed afterwards. The shell was removed manually after freeze-drying (Shimadzu Emit, Christ GDH-60, serial: 603,876). Further, the snails were imaged on a petri dish using a LED lamp as light source.

#### Analysis of physiological markers

The supernatant of prior bonded biofilm (described above) was removed with a custom-made glass U-tube. For two replicates of the exposure aquaria, 10 mg of wMWCNTs was weighed with a microbalance (analytical balance, Sartorius) and dispersed for 30 min among a continuous sonication (Bandelin Sonopuls GM 70, MS 72/0, P 60 W) in a beaker which contained 1 L of modified Borgmann medium. The dispersion was always recreated adequate for two replicates. Afterwards, 500 mL of the dispersion was added and carefully poured along the aquarium glass wall to each aquarium with bonded biofilm, and also, 500 mL of pure Borgmann medium was added to each control aquaria. The remaining dispersion in the beaker was sonicated again for 10 min to avoid agglomeration until it was exhausted. Following, five snails were put into each aquarium after adaption (see above) and equipped with a glass covering to avoid the creeping out. All aquaria were continuously renewed every 3 days during the whole effect study. Thereby, the snails were taken out of each aquarium and stored for a short time into a petri dish which was filled with Borgmann medium. After purification, the snails were returned into the new aquaria which were previously inhered each with benthic biofilm for 24 h. After 24 days, no wMWCNT was added to the exposure aquaria, and everything was renewed with pure Borgmann medium. Samples of *L. stagnalis* were randomly taken out and investigated after 24 days and additionally after 28 days of depuration.

As physiological markers, the concentration of glycogen and TGs and the ratio of RNA/DNA were investigated at the University of Koblenz-Landau. Four replicates from each sampling point, consisting of three snails respectively (pooled), were used for each physiological marker analysis. The shells of each individual were removed, and all samples were freeze-dried for 24 h (Shimadzu Emit, Christ GDH-60, serial: 603,876). Afterwards, the samples were stored in a desiccator until use. For each analysis, 2–3 mg of dried biomass of *L. stagnalis* was transferred into a pre-weighted 1.5 mL Eppendorf tube containing four glass beads. The weight was determined with a microbalance. Afterwards, the samples were homogenized in a bead mill (Retsch 40MM, Hahn, Germany) for 3 min at 25 Hz. The glycogen concentration was determined according to Koop et al. (2011). The extraction and quantification of TGs were conducted using the commercial TG assay DiaSys Diagnostic 2015 (Hoppeler et al. 2018). The RNA/DNA ratio was quantified via fluorometer (Qubit® 2.0 Fluorometer, Thermofisher, Waltham, USA) with the commercial assay MasterPureTM Complete RNA and DNA Purification (Epicentre, Madison, USA). For the extraction of nucleic acids, 1 mg dry tissue was homogenized in a bead mill by adding 300 μL lysis buffer (1 μL proteinase K, 300 μL tissue, and cell lysis solution) and incubated for 15 min at 65 °C in a thermomixer with 1200 rpm (Eppendorf Thermomixer comfort, Wesseking, Berzdorf, Germany). Subsequently, 150 μL of protein precipitation agent was added to each sample, which was then vortexed and centrifuged at 4 °C and 18,000 g for 10 min. After transferring the supernatant into RNase- and DNase-free Eppendorf tubes, 500 μL isopropanol was added which induced the precipitation of RNA/DNA. To facilitate precipitation, the samples were inverted several times and centrifuged at 4 °C and 18,000 g for 10 min. The pellet was rinsed twice with 70% ethanol and resolved in 50 μL TE-buffer. The concentration of RNA and DNA was measured with a plate reader (EnSpire Multimode Plate Reader, Perkin Elmer, Germany) using the Qubit™ dsDNA BR Assay Kit and Qubit™ RNA BR Assay Kit (Invitrogen™, Life Technologies™, Darmstadt, Germany). For the calculation of the lipid concentration in dry weight per pooled sample, the molar weight of the most common fatty acid in aquatic invertebrates, linoleic acid, was used (Arakelova et al. 2009). All the samples were determined in dry weight. For consideration in wet weight, a factor of 4.5 should be taken into account (Worischka et al. 2014).

#### Histology and electron microscopy

**Fig. 2:**
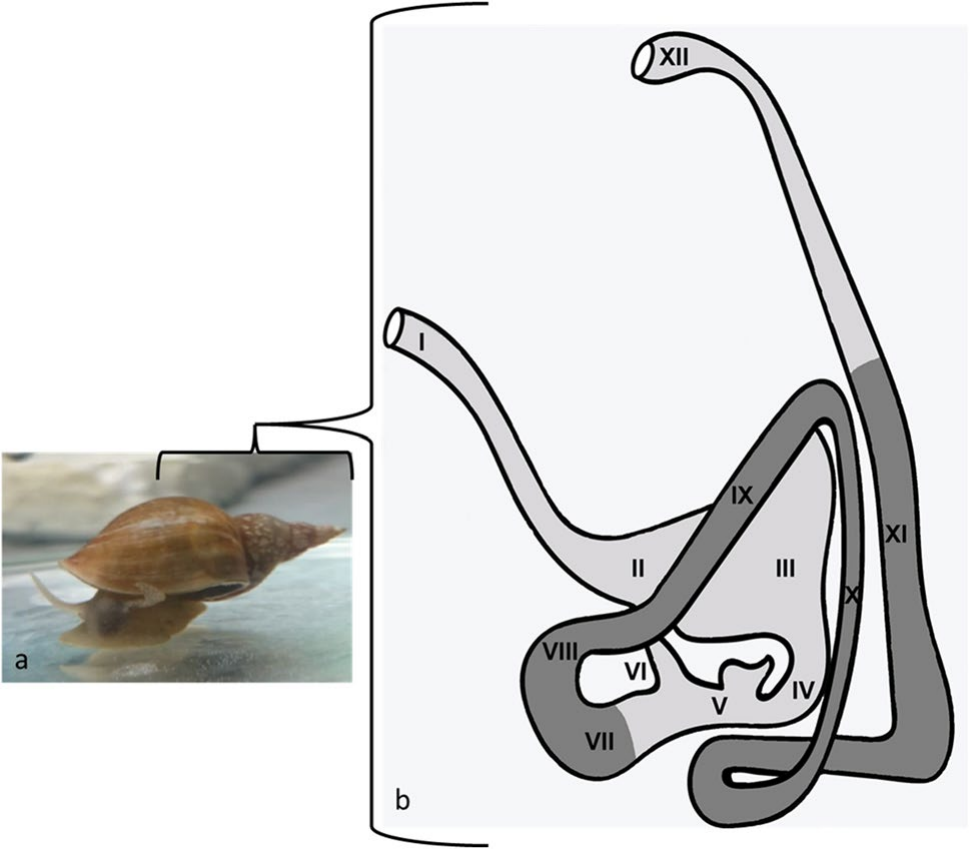
Depiction of *L. stagnalis* (**a**) and the associated sections of the alimentary tract (**b**). **I** Postesophagus, **II** crop, **III** gizzard, **IV** pylorus, **V** vestibulum with openings of the digestive gland ducts, **VI** cecum, **VII**–**IX** first to third part of prointestine, **X** midintestine, **XI** postintestine, and **XII** rectum. Investigated parts highlighted in grey (VII–XI). Reproduced according to Boer and Kits (1990)

For histology, samples of *L. stagnalis* were examined after 10 days of exposure. The intestinal tract of *L. stagnalis* and the investigated parts were highlighted in grey (Fig. 2, points VII–XI). Two of the five snails of control and exposition animals were used for histology investigations. For histology and EM, whole snails were sedated in 1% of hydroxylamine solution, followed by removal of the shell and fixation in 4% formaldehyde in 100 mM phosphate buffer. The dissected tissue from the digestive tract were postfixed in modified Karnovsky fixative (Karnovsky 1965, 2% glutaraldehyde, 2% formaldehyde prepared from PFA prills, 2 mM calcium chloride in 150 mM cacodylate buffer). After washing in cacodylate buffer and phosphate-buffered saline (PBS), the samples were decalcified in 20% aqueous EDTA (Osteosoft, Merck) for several hours at 37 °C, followed by washing in PBS and water. For histology, the samples were dehydrated in a graded series of ethanol/water mixtures (30%, 50%, 70%, 90%, 96%) up to 100% ethanol (2 ×) and infiltrated and embedded into the methacrylate resin Technovit 7100 (Heraeus Kulzer, see Kurth et al. 2012). Two-µm-thin sections were stained with toluidine blue/borax and analyzed with a Keyence Biozero 8000 light microscope. For SEM, the decalcified samples were postfixed with 1% osmium tetroxide, dehydrated in a graded series of ethanol (30%, 50%, 70%, 90%, 96%, 3 × 100% ethanol, pure ethanol on molecular sieve), and critical point dried using the Leica CPD300 dryer (Leica Microsystems, Vienna, Austria). Dried samples were mounted on a 12 mm aluminum stub coated with a conductive carbon pad and sputter coated with gold using the Baltec SCD 050 sputter coater (thickness 15 nm). Finally, the samples were analyzed with a Jeol JSM7500F cold field-emission scanning electron microscope (Jeol, Freising, Germany) at 5 kV acceleration voltage (working distance 8 mm, lower secondary electron detector). For TEM, the samples were postfixed and contrasted in 2% aqueous OsO_4_ solution containing 1.5% potassium ferrocyanide and 2 mM CaCl_2_ for 30 min on ice. After washing in water, the samples were incubated in 1% thiocarbohydrazide in water (20 min at room temperature), followed by washing in water and a second osmium contrasting step in 2% OsO_4_/water (30 min, on ice). Samples were washed in water and bloc contrasted with 1% uranyl acetate/water on ice overnight. Afterwards, it was washed again in water, dehydrated in a graded series of ethanol/water (30%, 50%, 70%, 90%, 96%, 3 × 100% ethanol, pure ethanol on molecular sieve), and infiltrated in the epoxy resin EMbed 812 (epoxy/ethanol mixtures: 1:3, 1:1, 3:1 for 1 h each, pure epon (epoxy resin) overnight, pure epon 5 h). Finally, the samples were embedded in flat embedding molds and cured at 60 °C overnight. After polymerization, the tissue was cut into semi-thin sections of 1 μm with a glass knife using the Leica UC6 ultramicrotome (Leica Microsystems, Wetzlar, Germany). The sections were stained with 1% toluidine blue and 0.5% borax to evaluate the tissue quality and select areas of interest. Afterwards, ultrathin Sects. (70 nm) were prepared, collected on formvar-coated slot grids, and stained with lead citrate (Venable and Coggeshall 1965) and 4% uranyl acetate. Contrasted ultrathin sections were analyzed on a FEI Morgagni D268 (FEI, Eindhoven, The Netherlands, camera: MegaView III, Olympus) or a Jeol JEM1400 Plus (JEOL, Garching, Germany, camera: Ruby, JEOL) both at 80 kV acceleration voltage.

## Statistics

Data analysis was performed with software R (RStudio Team 2017). The statistical test is a Wilcoxon signed-rank test, which is applicable for outlier analysis and thus resistant to aberrations. This nonparametric test was used because of the small sample size. The significance level was *p* ≤ 0.05 with the corresponding *W*-value (test statistic). A decrease in the concentrations of the physiological marker was expected in the experiment. Therefore, a one-sided test was applied. For the controls, however, no direction was expected. Hence, a two-sided test was realized.

## Results

### Quantification study

#### Quantification on ^14^C-wMWCNT uptake in benthic biofilm and *L. stagnalis*

**Fig. 3:**
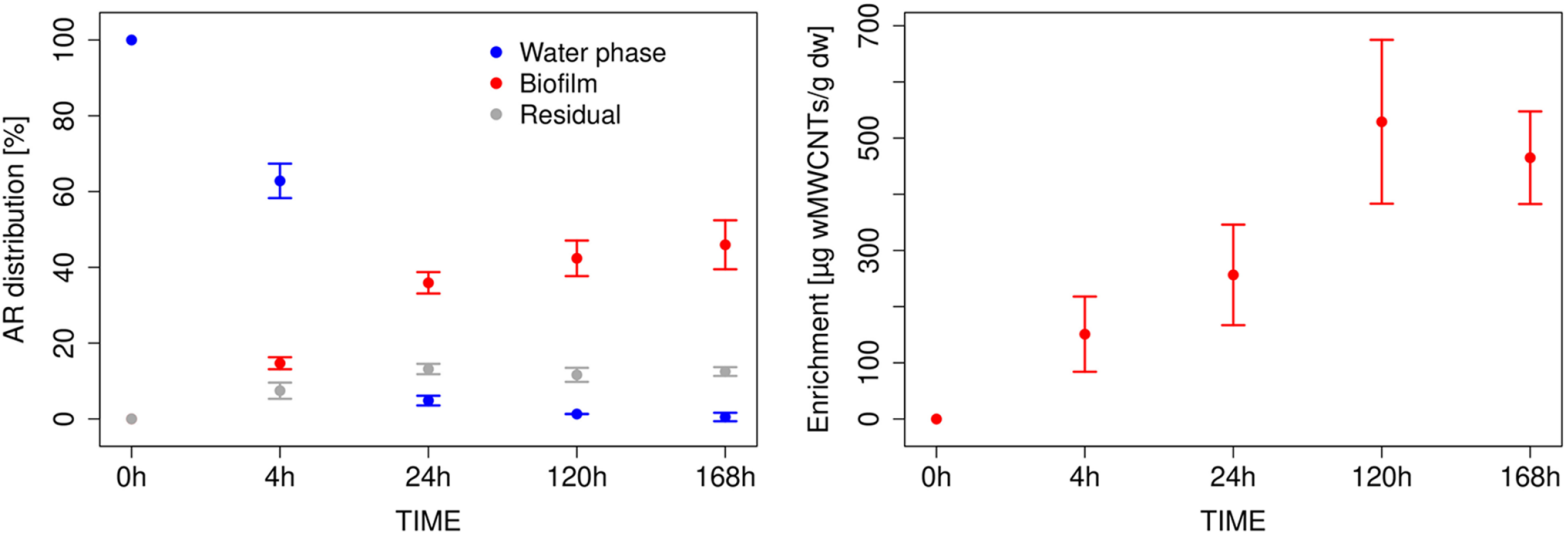
Left: relative distribution: [%] of AR in the different phases of the experimental setup with benthic biofilm. Shown are the water phase (blue dots), biofilm (red dots), and residuals (grey dots) with mean. Right: enrichment of the ^14^C-wMWCNTs in the benthic biofilm over time with mean and relative standard deviations. Note the nonlinear time axis

For all control samples concerning benthic biofilm and *L. stagnalis*, no radioactivity was detected. The distribution of radiolabeled ^14^C-wMWCNTs between water and biofilm was investigated (Fig. 3, left). The radioactivity decreased in the water phase over time. From the beginning of the experiment with 100% (t0), equal to a concentration of 0.1 mg wMWCNT per liter, the amounts declined to a value of 62.8 ± 4.5% (4 h) in the water phase. Afterwards, 4.8 ± 1.3% (24 h), 1.28 ± 0.04% (120 h), and 1.72 ± 1.1% (168 h) of applied radioactivity (AR) was found in the water phase. Thus, a noticeable decrease in ^14^C-wMWCNT concentration was recorded, while the radioactivity in the benthic biofilm increased from 14.7 ± 1.6% (4 h) to 35.9 ± 2.8% (24 h), 42.4 ± 4.7% (120 h), and 45.9 ± 6.5% (168 h) (Fig. 3, left). The residuals as mentioned above amounted to 7.4 ± 2.4% (4 h), 13.2 ± 2.1% (24 h), 11.6 ± 2.2% (120 h), and 12.5 ± 1.4% of AR at the end of the experiment. The recovery rate of ^14^C-wMWCNTs for each sampling point is the sum of the relative amount of water phase, biofilm, and residuals. Consequently, the recovery declined from 85 ± 2.4% (4 h) to 60.2 ± 5.3% (168 h); i.e., a loss of approximately 40% of AR was found at the end of the experiment. The maximum uptake of ^14^C-wMWCNT in benthic biofilm was reached after day 5 and amounted to 529 µg wMWCNT/g dry weight (Fig. 3, right). Afterwards, the value decreased slightly to 465 µg wMWCNT/g dry weight at day 7. No *BCF* was calculated for benthic biofilm because no separation of wMWCNTs from the complex biofilm suspension was possible.

**Fig. 4:**
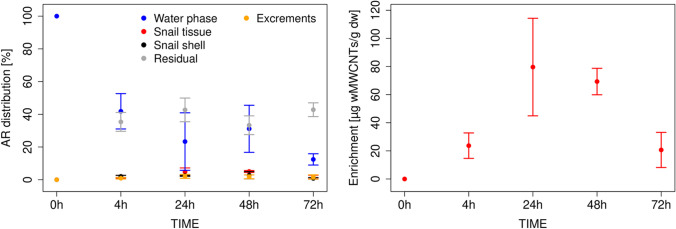
Left: relative distribution: [%] of AR in different compartments are shown as the water phase (blue dots), snail tissue (red dots), snail shell (black dots), excrements (yellow dots), and residuals (grey dots) with mean of the experimental setup for *L. stagnalis* [%]. Right: enrichment of the ^14^C-wMWCNTs in *L. stagnalis* tissue between time with mean and relative standard deviation

Relative amounts of ^14^C-wMWCNTs in snail tissue ranged from 1.9 ± 0.8% (4 h), 4.8 ± 2.4% (24 h), 5.0 ± 0.6% (48 h) to 1.6 ± 1.1% (72 h) (Fig. 4, left). Additionally, ^14^C-wMWCNTs for the snail shell was found with results of 2.0 ± 0.7% (4 h), 2.4 ± 0.4% (24 h), 2.7 ± 2.2% (48 h), and 0.8 ± 0.5% (72 h). The excrements showed a percentage radioactivity of 0.9 ± 0.4% (4 h), 2.0 ± 1.0% (24 h), 1.8 ± 1.0% (48 h), and 1.7 ± 1.1% (72 h). A same course for the parts of snail tissue, snail shell, and excrements of *L. stagnalis* is discernible. The residuals that included the cellulose cloth and Pasteur pipettes feature values of 35.4 ± 2.0% (4 h), 42.7 ± 2.7% (24 h), and 33.3 ± 2.2% (48 h). At 72 h, the cover net of the aquaria was added to the whole residual phase, and thus, a higher radioactivity than for the other sampling points was analyzed (47.0 ± 3.5%). The recovery again was low ranging from 82 ± 4.2% (4 h) to 60.2 ± 5.3% (72 h). The enrichment of ^14^C-wMWCNT in *L. stagnalis* shows a maximum of 79.6 ± 34.7 µg wMWCNT/g dry weight at 24 h (Fig. 4, right), but decreased to 20.65 ± 12.50 µg wMWCNT/g dry weight (72 h). Considering the large scatter of data with standard deviations at 48 h and 72 h of 9.4% and 9.0%, respectively, the accumulation of ^14^C-wMWCNTs in *L. stagnalis* could have reached steady state. ^14^C-wMWCNTs continuously accumulate in the benthic biofilm after more than 24 h (Fig. 3, right), whereas a steady state of ^14^C-wMWCNTs uptake in *L. stagnalis* was achieved at 24 h (Fig. 4, right).

The relative distribution of ^14^C-wMWCNTs in *L. stagnalis* (Fig. 4, left) shows a decrease of ^14^C-wMWCNTs in the water phase over time. The amounts of ^14^C-wMWCNTs in the water phase ranged from 41.8 ± 9.4% (4 h), 23.3 ± 15.2% (24 h), 31.1 ± 12.5% (48 h) to 12.4 ± 3.0% (72 h).

Due to the steady state in *L. stagnalis*, a *BCF* of 3500 L/kg in tissue was ascertained, and the results of all *BCFs* in tissue dry weight per time points are shown in Table 2.

**Table 2:** The bioconcentration factor [L/kg] and log (*BCF*) of ^14^C-wMWCNTs over time in tissue of *L. stagnalis*

Time	***BCF*** tissue dry weight	Log (***BCF***)
4 h	510	2.7
24 h	3481	3.5
48 h	2254	3.4
72 h	1452	3.2

### Effect study

#### TEM and SEM investigation of MWCNTs, wMWCNTs, and benthic biofilm

**Fig. 5:**
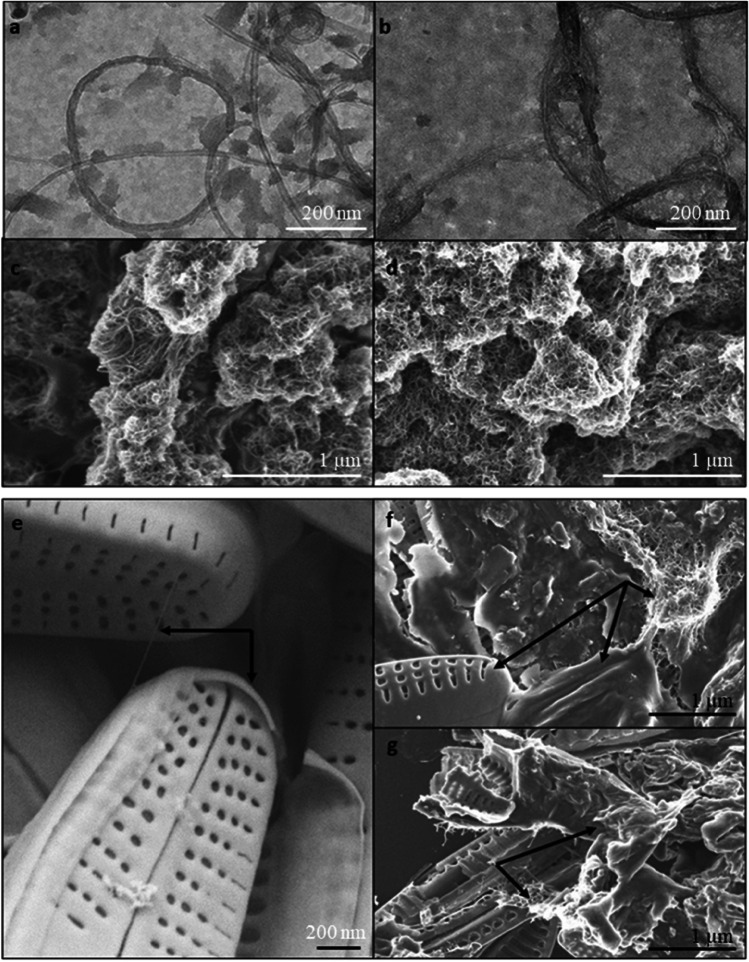
TEM (**a**–**b**) and SEM (**c**–**g**) images of wMWCNTs (**a**, **c**), MWCNTs (**b**, **d**), and benthic biofilm after 1 week of exposure to 0.1 mg/L wMWCNTs (**e**–**g**) with **e** diatoms with an interacting EPS among themselves and **f**–**g** diatoms with wMWCNTs and EPS interacting network

Structural insights by electron microscopy (TEM, SEM) of wMWCNTs, MWCNTs, biofilm, and extracellular polymeric substances (EPS) are given in Fig. 5. Figure 5a and b shows the wMWCNTs and MWCNTs in TEM and Fig. 5c and d in SEM. The structures of wMWCNTs and MWCNTs for TEM are similar and look like helices with irregularities in their form. The SEM images feature a complex entangled structure of both (Fig. 5c, d). No structural differences are visible. Biofilm was exposed for 1 week to wMWCNTs and was imaged with SEM (Figure, 5e–g). Figure 5e reveals an interaction between diatoms among themselves without wMWCNT (Fig. 5e, arrows). After exposure to wMWCNTs, a more complex network is apparent, and in some areas, a network between extracellular polymeric substances (EPS) of some broken diatoms and wMWCNTs was observed, too (Fig. 5f–g, arrows). It seems to be a conjunction between diatom cells and their EPS together with wMWCNTs (Fig. 5f–g).

#### Visual examination of *L. stagnalis*

**Fig. 6:**
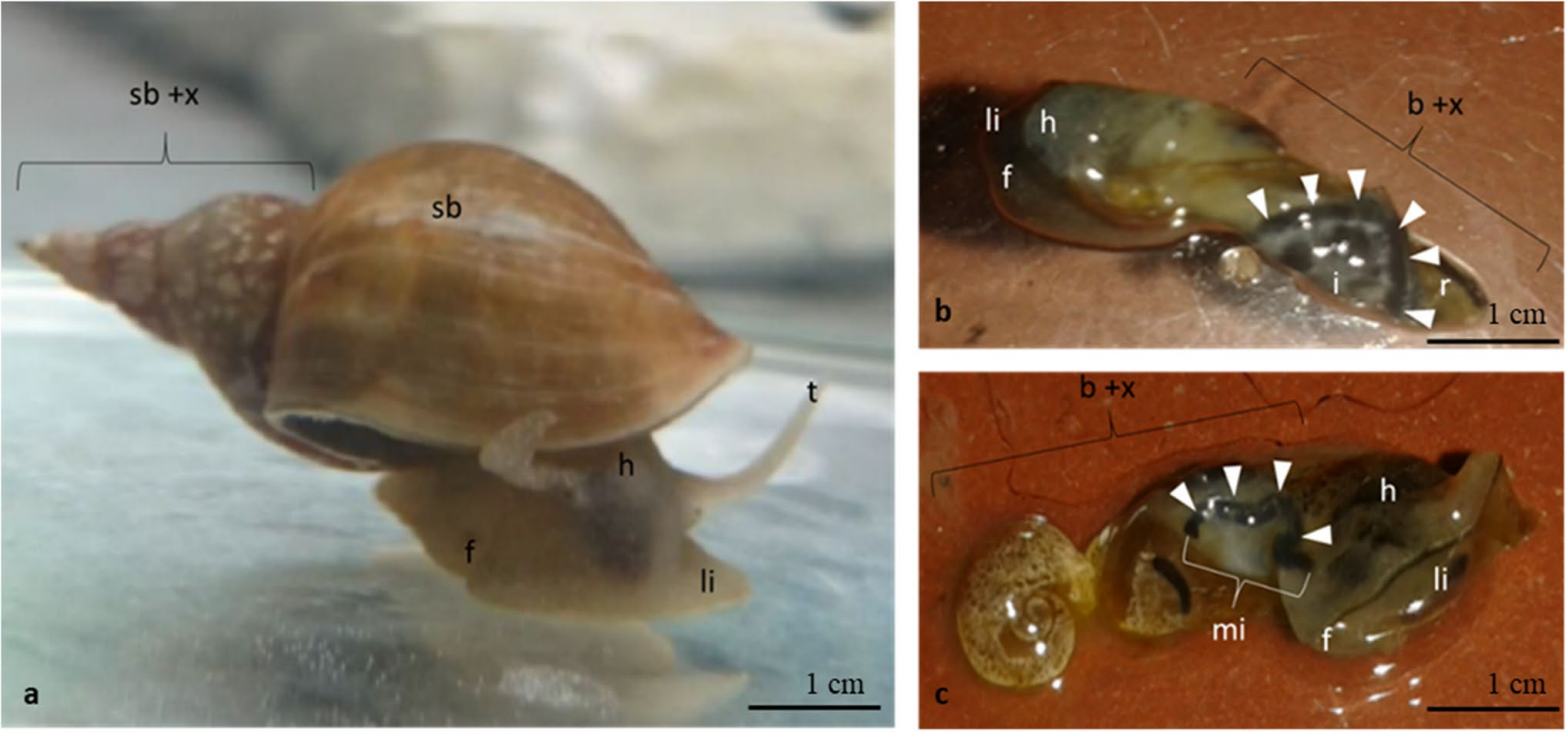
Pictures of *L. stagnalis* from control (**a**) and exposure (10 mg/L wMWCNTs) treatment after 1 week (**b**–**c**). **a** sb, shell-body; sb + x, parts of the crop to the rectum (see also Fig. 2); h, head; t, tentacle; f, foot; li, lips. The control is represented as whole individuum with the part sb + x as the investigated area between the crop until the rectum where the intestine is in between. The treatments (**b**–**c**) without shell where the intestine is visible as a black line (white arrowheads). **b** b + x, body without shell (parts from the crop to the rectum); i, intestine. **c** b + x with additionally mi, midintestine

To demonstrate the accumulation of wMWCNTs by *L. stagnalis*, the individuals were dissected from their shells and imaged (Fig. 6a–c). The control sample with shell (Fig. 6a) exhibits a brown hue. The contaminated samples (Fig. 6b–c) illustrate strong grey staining from the head up to the intestine. It is discernible that the intestine is completely black (Fig. 6b). The example in Fig. 6c shows a black midintestine. In contrast to this, no grey tinge of these parts was detected in the control individuals; rather, the whole body appears tanned (Fig. 6a).

#### Analysis of physiological markers

**Fig. 7:**
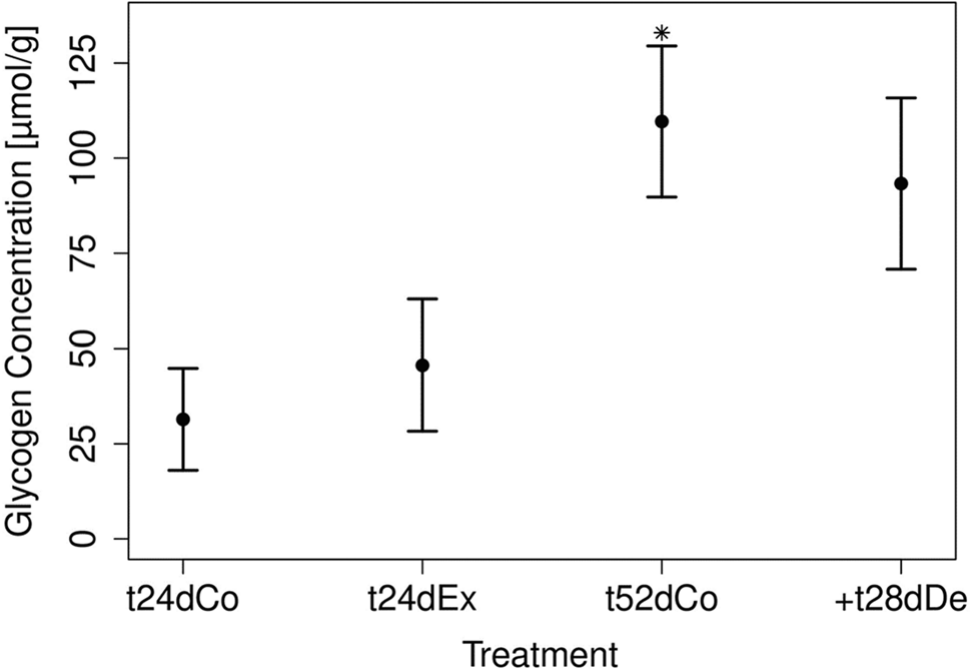
Results of the glycogen analysis after 24 days and 52 days with mean and relative standard deviation. The controls are depicted after 24 days as t24dCo and after 52 days as t52dCo. The exposures are depicted after 24 days with wMWCNT as t24dEx and depuration after 28 days as + t28dDe, p* = 0.03, *n* = 4

**Fig. 8:**
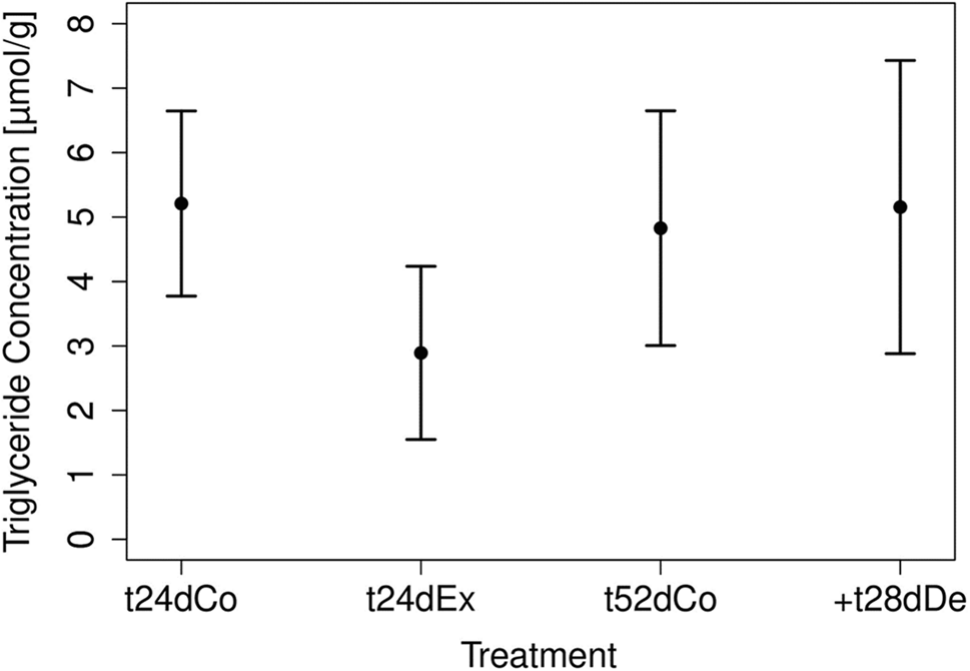
Results of the TG analysis after 24 days and 52 days with mean and relative standard deviation. The controls are depicted after 24 days as t24dCo and after 52 days as t52dCo. The exposures are depicted after 24 days with wMWCNT as t24dEx and depuration after 28 days as + t28dDe, *n* = 4

**Fig. 9:**
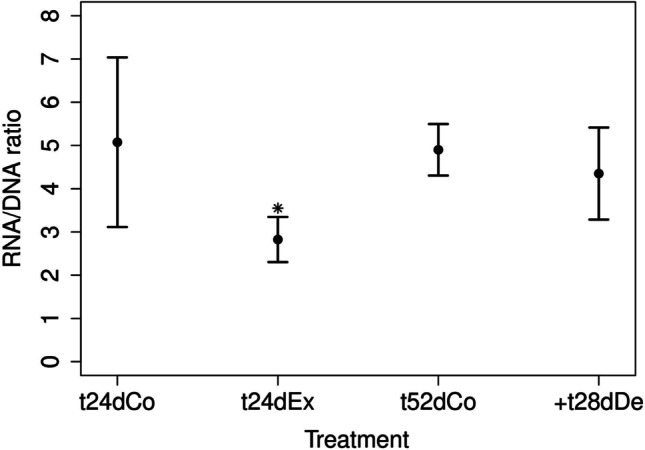
Results of the RNA/DNA analysis after 24 days and 52 days with mean and relative standard deviation. The controls are depicted after 24 days as t24dCo and after 52 days as t52dCo. The exposures are depicted after 24 days with wMWCNT as t24dEx and depuration after 28 days as + t28dDe, p* = 0.01, *n* = 4

The concentrations of the physiological marker glycogen and TGs as well as RNA/DNA ratios in the control and exposed snails were investigated (Figs. 7–9). In the following, the time point “24dEx” denotes the measured quantity after 24 days of exposure, and “ + 28dDe” corresponds to the treatment where the formerly exposed snails have undergone a depuration phase of 28 days afterwards. For comparison, untreated animals were examined as a control group also after 24 days (“24dCo”) and 24 + 28 = 52 days (“52dCo”). For glycogen (Fig. 7), the control shows a significant increase over time between t24dCo (mean: 31.43 µmol/g dry weight) and t52dCo (mean:109.64 µmol/g dry weight, Table 3). However, after t24dEx, the median value did not differ significantly from the control t24dCo (Table 3). No significant difference was found at the end of the experiment between the control t52dCo and the exposure approach past depuration + t28dDe with a mean of 93.31 µmol/g dry weight.

**Table 3:** Statistical analysis of the quantitative values for glycogen, TGs, and RNA/DNA among different time points, and the corresponding *W* and *p* values are shown

W and *p* values	Glycogen	TGs	RNA/DNA
W(t24dCo/t52dCo)	0	10	5
*p*(t24dCo/t52dCo)	0.03	0.69	0.49
W(t24dCo/t24dEx)	4	13	16
*p*(t24dCo/t24dEx)	0.9	0.10	0.01
W(t52dCo/ + t28dDE)	10	7	11
*p*(t52dCo/ + t28dDE)	0.34	0.66	0.24

The TGs and lipid concentrations were similar over the experimental duration in the controls (mean of t24dCo: 5.21 µmol/g and 1.46 mg/g dry weight of lipid concentration; mean of t52dCo: 4.83 µmol/g and 1.35 dry weight of lipid concentration, Table 3, Fig. 8). The control t24dCo compared to the exposure t24dEx (Fig. 8) features no significant differences between them, but it can be interpreted as weakly significant within a significance level of 10% (Table 3). Among t52dCo and + t28dDe, no significances in TGs concentration are analyzed (Table 3). After the depuration, 1.45 mg/g dry weight and thus a similar value to both controls (t24dCo and t52dCo) was calculated for the lipid concentration.

There was no difference in RNA/DNA analysis between controls over time (mean of t24dCo: 5.1, mean of t52dCo: 4.9), whereas the ratio was significantly reduced in t24dEx (mean 2.8) compared with t24dCo (Table 3, Fig. 9). After + t28dDe, the previously exposed animals reached the same level of RNA/DNA ratio as the group of animals that had never been subjected to wMWCNTs (mean 4.4 and 4.9, Table 3, Fig. 9).

#### Histology and electron microscopy of snail intestine

**Fig. 10:**
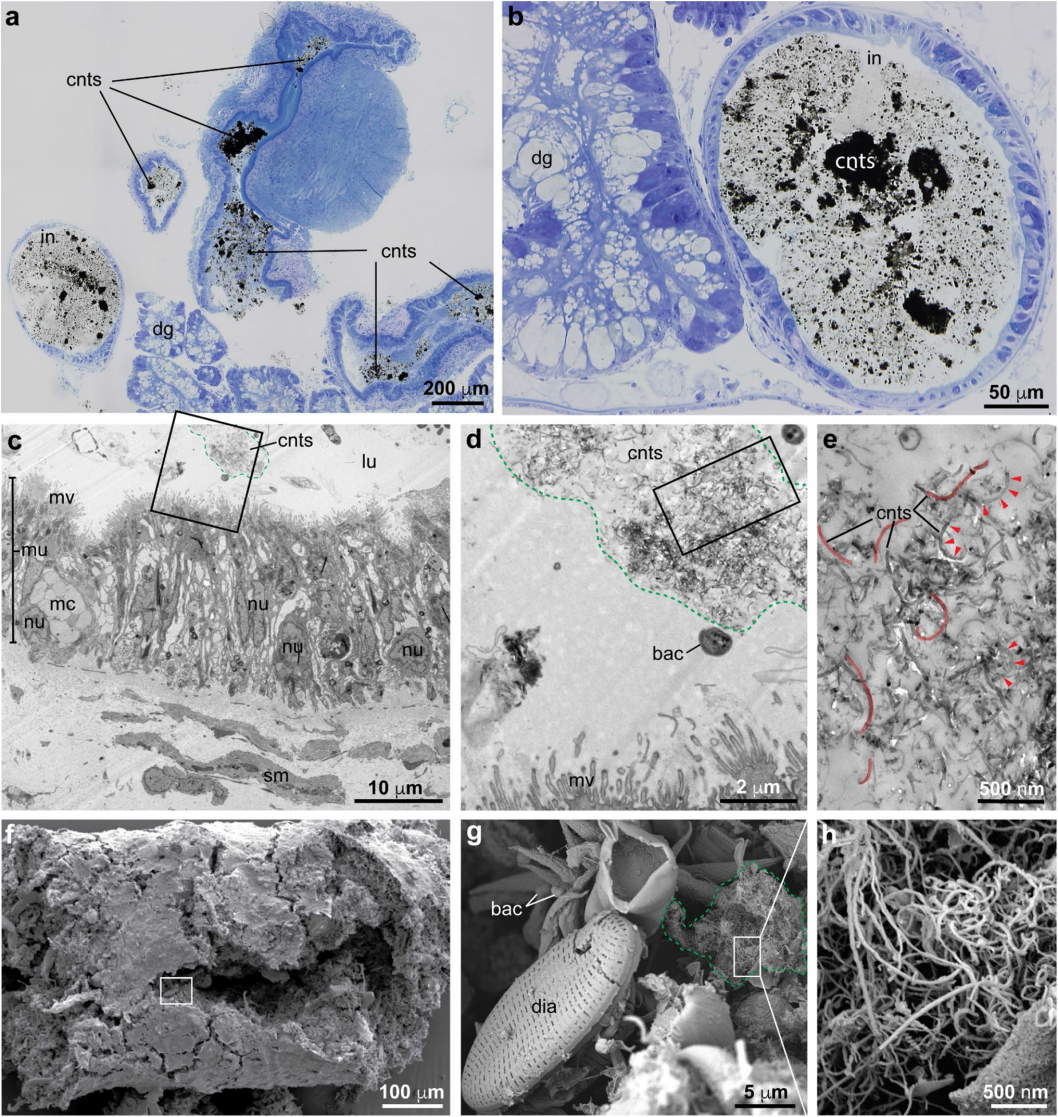
Histology and electron microscopy of wMWCNTs in the intestinal tract of *L. stagnalis*. **a**, **b** Toluidine blue/borax-stained methacrylate resin sections. An overview of different parts of the intestinal tract with wMWCNTs in the lumen (cnts). **b** Higher magnification of the intestine (in) and adjacent digestive gland (dg). Bundles of wMWCNTs are clearly visible as black masses in the lumen of the intestinal tube. **c**–**e** TEM of ultrathin epon sections through the intestinal wall. **c** Overview with lumen (lu), mucosa (mu), and underlying submucosa with smooth muscle cells (sm). A bundle of CNTs is visible in the lumen. The square indicates the region shown in **d**. **d** the CNT bundle at higher magnification in the vicinity of the apical microvilli of the enterocytes. The square indicates the region shown in **e**. **e** CNTs from **d** shown at higher magnification. Some CNTs are indicated by arrowheads, and some are color coded in red (30% opacity). **f**–**h** SEM. **f** Intestinal content that was squeezed out of the intestinal tube. **g** Intestinal content containing bacteria, algae (diatom, dia), and CNTs (surrounded by a green dashed line). The squares in **f** and **g** indicate the regions magnified in **g** and **h**, respectively. **h** CNTs at high magnification. Further abbreviations: mc, mucus producing cell (goblet cell); nu, nucleus

To further understand the impact of the wMWCNTs on the snails, we took a closer look to potential contact areas between the snail body and the wMWCNTs (cnts), focusing on the intestinal tract. The wMWCNTs are ingested together with the biofilm, and histology of whole snails demonstrated them in the lumen of the complete intestinal tract (e.g., in the esophagus, stomach, and different parts of the intestine, Fig. 10). wMWCNTs are recognizable as black accumulated material in the lumen of the digestive tract (Fig. 10a, b). To further characterize the accumulated wMWCNTs, we dissected pieces of the intestine (Fig. 2, regions VII–XI) and processed them for electron microscopy. The tissue was either embedded in epon and ultrathin sections were prepared for TEM (Fig. 10c–e) or they were dehydrated and critical point dried for SEM (Fig. 10f–h). The wMWCNTs can be identified as thick bundles (indicated by the dashed green lines in Fig. 10c and d) that are found together with bacteria and algae in the intestinal lumen, close to the apical microvilli of the enterocytes (Fig. 10d). At higher magnifications, the wMWCNTs appear as sharply outlined tubes with open ends (Fig. 10e). Their characteristics are the unique shape and refracting properties, allowing a differentiation between wMWCNTs and other organic structures, such as membrane fractions (see Fig. 10e). In the SEM, the content of the intestine shows a typical biofilm composition including diatoms and bacteria (Fig. 10f, g). The wMWCNTs again are apparent as bundles of fibrous material (indicated by the green dashed line in Fig. 10g) equivalent to what is obvious in TEM images. At higher magnification, wMWCNTs can be distinguished by their typical structure (compare Fig. 10h to Fig. 5a). Closer inspection of the enterocytes, however, revealed no intracellular wMWCNTs (images not shown). Uptake of the wMWCNTs occurs only in the digestive glands (not shown).

## Discussion

We set up a workflow to study the impacts of wMWCNTs on species, communities, and trophic interactions in an aquatic ecosystem addressing multiple endpoints. As a model system, we took *L. stagnalis*, a grazing pulmonary mud snail with a crucial role in the aquatic food web. We choose different parameters to address the accumulation and behavior of wMWCNTs in biofilms and snails using radioactively labeled wMWCNTs, physiological markers, and microscopical techniques. To our knowledge, no comparable data is available for experiments with ^14^C-wMWCNTs in biofilms or for histology in *L. stagnalis*. Because of the high variety of concentrations of CNTs generally occurring in the environment, we used different concentrations in our experiments to cover various scenarios, ranging from 0.1 mg/L ^14^C-wMWCNTs to 10 mg/L in the effect studies. This approach is also applicable to other aquatic indicator organisms and may provide a useful pipeline for the thorough analysis of the effects of nanomaterials on aquatic ecosystems. The survival, development, and response of control organisms in our approach met the validity criteria in accordance with the OECD (2016) guideline no. 243, and no mortality in the control groups as well as for wMWCNT-exposed individuals of *L. stagnalis* was demonstrated in all studies. We were able to show interacting diatoms and detected no obvious damages of species in the absence of wMWCNTs. The controls for TGs and RNA/DNA reveal a good survival rate and the individuals fared well. Eminently, the increase of the glycogen content in the controls shows adaptation to the given system. In summary, the results and the conformity among the different controls means no impairments by the experimental test conditions including the used Borgmann medium.

### Quantification of uptake and elimination of ^14^C-wMWCNTs in benthic biofilm and in *L. stagnalis*

Using radioactive labeling, we were able to show that wMWCNTs accumulate in benthic biofilm. The enrichment in benthic biofilm is significantly higher compared to the uptake in snails. The biofilm as a surface-covering layer in the aquarium offers a large surface area for the accumulation of wMWCNTs acting as a sink, and that might explain the difference. Calculating a *BCF* for benthic biofilm is difficult due to its composition of diverse organisms. Bjorkland et al. (2017) provided a tabular overview of different *BCFs* for single primary producers and ^14^C-MWCNTs. For example, the algae *Desmodesmus subspicatus* (*D. subspicatus*) was exposed to 1 mg/L ^14^C-MWCNTs for 72 h. A *BCF* of 5000 L/kg was determined (Rhiem et al. 2015). For the ciliate *Tetrahymena thermophila* (*T. thermophila*) exposed to 0.3 mg/L acid-purified ^14^C-MWCNTs for 22 h, a *BCF* of 800 L/kg was calculated (Mortimer et al. 2016). However, these factors were obtained in experiments with single species. In contrast, the biofilm community comprised a complex system of different groups of organisms. Differentiation of the radiolabeled wMWCNTs in the various fractions of the biofilm is not possible; thus, we did not calculate a *BCF*.

For *L. stagnalis*, a *BCF* of 3500 L/kg was ascertained in our study. Petersen et al. (2009) calculated normalized body burden values of 440,000 ± 190,000 (equivalent to *BCF*) for *Daphnia magna* during 48 h with 0.1 mg/L acid-oxidized ^14^C-MWCNT dispersed in water. Bjorkland et al. (2017) reported in their summary a *BCF* of 73 L/kg after 7 days exposure to 1 mg/L acid-purified ^14^C-MWCNTs to *Danio rerio* (Maes et al. 2014a). For comparison, *D. magna* is a crustacean and able to breathe through the surface of their turgor extremities and through their entire body surface. Thus, the high *BCF* of daphnids could be explained in comparison to our results and to the results of Maes et al. (2014a). These different bioaccumulation factors are attributed to the different ingestion routes and sizes of the respective organisms, but also due to the chemical structures of the test substances. Lalah et al. (2003) exposed *L. stagnalis* to ^14^C-p-nonylphenol. They observed the highest BAF (described as it refers to *BCF*) of 242 L/kg after 3 days of exposure. Although a comparison of non-soluble nanomaterial and a dissolved organic compound regarding uptake is difficult, it reflects the accumulation potential of the snails.

In general, to investigate a *BCF* in a test system with *L. stagnalis* is difficult because the snails continuously move all over the aquarium and can accumulate via water and via food. The radioactivity in the water phase decreased rapidly due to sedimentation and agglomeration of the nanotubes. Concomitantly, the recovery of radioactivity decreased up to a loss of ca. 40%. We suspect that due to the size of the aquarium in our experiments, and the high affinity of the nanotubes to adsorb on surfaces such as glass walls and seals, some of the radioactivity could not be recovered. Petersen et al. (2009) explained a low recovery of about 60% in his experiments assuming a similar reason.

### Glycogen, triglycerides, and RNA/DNA ratio as physiological markers in *L. stagnalis*

We showed that wMWCNTs certainly have an influence on anabolic processes, most likely somatic growth, because the RNA/DNA ratio was significantly reduced upon exposure to wMWCNTs. It is a common hypothesis that the RNA/DNA ratio increases during periods of high metabolic activity and decreases in the case of growth inhibition. High metabolic activities might occur during moderate stress situations. Consequently, organisms have high maintenance costs due to the compensatory upregulation of the detoxification and cell protection mechanisms (Sokolova et al. 2012). The RNA/DNA ratio provides information about the actual investment and the associated anabolic activity of organisms. Elser et al. (2005) found a RNA/DNA ratio of 4.3 in their control snails (*Mexithauma quadripaludium*) in a study on the response to phosphorus enrichment by modern stromatolitic microbial communities. Our results showed similar RNA/DNA ratios for the controls.

The ratio has often been used as an indicator of the nutritional status of fish larvae and larval growth (Buckley 1984; Peck et al. 2003). This statement is supported by the observation that the ratio in our experiments increases again after depuration. Thus, it indicates some toxic stress for the organisms after exposure, but they seem to partially recover.

The analysis of physiological markers such as the glycogen and TGs is a suitable tool to investigate environmental impacts on animals (Koop et al. 2008; Zubrod et al. 2011). Energy storage is a process when energy intake exceeds current energy demand, thus enabling survival during periods of food shortage. In addition, stressors such as chemical pollutants can cause higher energy demands and thus lead to changes in the energy metabolism. Decreased energy stores might be interpreted as an indicator of moderate physiological stress (Sokolova et al. 2012). Alterations in the energy budgets can lead to changes in macronutrient contents (Nisbet et al. 2012; Fidder et al. 2016). In our experiments, exposure to wMWCNTs did not significantly change the glycogen concentration. Mohamed and Geraerts (1976) reported a degradation of glycogen during the transition from aerobiosis to anaerobiosis. In the absence of food, they detected 86.4 µmol/g wet weight (19.2 µmol/g dry weight) of glycogen after 24 h in control individuals. Changing from aerobic to anaerobic conditions after 15 h, the glycogen level dropped to 71.4 µmol/g wet weight (15.9 µmol/g dry weight) and 59.5 µmol/g wet weight (13.2 µmol/g dry weight). The glycogen concentrations in our control groups were between 15.8 and 48.9 µmol/g dry weight at 24 days and increased at 52 days (87.6–140.3 µmol/g dry weight). The reason for that could be increased glucose storage (Melendez et al. 1997) representing a snapshot of the energy level. A further reason for the increased glycogen concentration could be the different developmental stage of the individuals investigated at that day.

The TG level is considered to reflect the long-term status. McCauley et al. (1990) described the lipid concentration as an indicator of toxic effects and/or organismal performance. Reategui-Zirena and Salice (2018) reported differences in the concentration of nutrients in the offspring of *L. stagnalis* affected by cadmium, pyraclostrobin, and tributyltin. They measured among others the lipid concentration in egg masses and adult snails. They state that the lipids are suitable indicators for the nutritional state in cladocerans. For gastropods, less data are available for comparison (Reategui-Zirena and Salice 2016). We calculated a lipid concentration of 0.15% dry weight in the controls and 0.08% in the exposure individuals at 24 days. After the depuration, 0.14% was found in lipid concentration and thus similar to the control. Lalah et al. (2003) analyzed a lipid concentration of 4.4% wet weight (0.96% dry weight) after 3 days of exposure to ^14^C-p-nonylphenol isomer assuming a steady state of accumulation in *L. stagnalis* (size: 2.92–5.05 cm). In our experiments, we noticed a decrease of TGs and lipid concentrations. Following the depuration, the mollusks were able to regain 98.9% of the lipid concentration, and thus, they recover their physiological state.

### Distribution of wMWCNTs in the intestinal tract

Our histological analysis showed that wMWCNTs pass the digestive system and can be detected in the lumen as bundles from the esophagus to the rectum. They form black agglomerates in the digestive system. Jugdaohsingh et al. (1998) found a high affinity for polyhydroxy aluminum to mucus by grazing contaminated biofilm, but it was also found in the digestive system of *L. stagnalis*. The wMWCNTs can be recognized as agglomerate piles located extracellularly in front of the microvilli of the enterocytes. After depuration, no wMWCNTs were found extracellularly in the digestive system. The wMWCNTs accumulate not just everywhere but mainly in the digestive tract. Clearly visible accumulated wMWCNTs are found in the midintestine. The quantification study gives a view of the ability to enrich the ^14^C-wMWCNTs in a moderate way, and microscopy reveals a semiquantitative analysis of their local distribution. Amorim et al. (2019) feature a compilation of classical toxicological values and of bioaccumulation in *L. stagnalis*. They summarized previously published ecotoxicological tests with *L. stagnalis*. There are 28 publications concerning tissue analyzing of digestive glands as described in Elangovan et al. (1997), Coeurdassier et al. (2004), Dobranskyte et al. (2004, 2006), or, e.g., Walton et al. (2009). Carriker and Bilstad (1946) disclosed the swallow of organic and inorganic materials in digestive cells in *L. stagnalis*. Further on, small and indigestible particles (0.1–0.4 µm) can be incorporated into small, green, and yellow granules, which are located in the digestive gland (Walker 1970). Elangovan et al. (2000) investigated the fate and localization of aluminum in the digestive gland of *L. stagnalis*. The electron microscopy showed different granule types. This reflects the digestive gland as a sink of accumulated aluminum. These granules are grain-shaped storages in biological cells, which include storage substances like glycogen, lipid, or protein.

### Trophic transfer of wMWCNT

An influence on benthic biofilm as the food source for lots of primary consumers has been depicted in the EPS interaction with wMWCNTs by SEM. EPS provide a matrix for embedding microorganisms in the biofilm and are able to keep agglomerates in their three-dimensional arrangement (Schulte and Flemming 2006). Even low concentrations of 0.1 mg/L wMWCNTs had an influence on the biofilm expressing a wMWCNT-EPS network and possible further growth inhibition. We found out that *L. stagnalis* was grazing the whole biofilm with its radula. Garacci et al. (2016) observed a similar network of graphene oxide layers and EPS of the freshwater diatom *Nitzschia palea*. In comparison to the experiment of Garacci et al. (2016), we obtained similar results of network formation already at a much lower concentration of 0.1 mg/L wMWCNTs after 1 week. In contrast, we investigated a natural biofilm composition and observed the kind of nanotubes amalgamated in this matrix. Thus, probably, a transfer of wMWCNTs through the higher trophic levels occurs.

Studies of Petersen et al. (2008) and Parks et al. (2013) displayed no negative effects on organisms exposed to nanoparticles (NP), whereas our results reveal obvious negative effects. Other studies with NP also revealed impacts on different organisms in vitro and in vivo (Werlin et al. 2011; Dong et al. 2018; Su et al. 2018; Griffit et al. 2011). Werlin et al. (2011) investigated the effect of accumulated cadmium selenide quantum dots in *Pseudomonas aeruginosa*, when transferred into the ciliate *T. thermophila.* Afterwards, the concentration of quantum dots in the predator *T. thermophila* was five times higher as for the bacterial loot. Thus, quantum dots can be bioavailable for higher trophic levels in the food chain. Additionally, Dong et al. (2018) explained the graphene materials as a grown industry and investigated the uptake of ^14^C-labeled graphene from *Escherichia coli* to *T. thermophila*. They calculated a high trophic transfer factor (TTF) from 0.2 to 8.6 and, thus, a high potential in the aquatic food chain. Contrary, from *T. thermophila* to *D. rerio* revealed a TTF lower than 1. That implies how different the trophic transfer can be through the food chain. Further on, Su et al. (2018) investigated the uptake of ^14^C-labeled few-layer graphene (FLG) in freshwater snails. They concluded a significant higher accumulation of FLG in the presence of algae than in the absence. Afterwards, the FLGs permeate through the intestinal wall and to the intestinal epithelial cells. Griffit et al. (2011) investigated the chronic effects of silver nanoparticles (AgNP) to juvenile and adult sheepshead minnows *Cyprinodon variegarus*. Low amounts of AgNP showed a significant tissue burden for juveniles and adults which means a thickening of the tissue of epithelia gills. Likewise, Daoud et al. (2014) investigated the acute toxicity and genotoxicity of AgNP to the freshwater snail *L. luteola* for 96 h and explained that the toxicity of AgNP depends on many factors like the size, surface area, and chemical compositions.

Hudson et al. (2019) explained the trophic transfer of gold nanoparticles to be organism specific in aquatic food webs. This was attributed to the unique feeding mechanisms of species. Balog et al. (2012) and Feiyue et al. (2004) qualified *L. stagnalis* as a more general feeder removing the complete periphyton matrix with its radula. In contrast, *Hyalella azteca* grazes rather selectively from the bottom side of the tiles where no periphyton had grown in the experiments (Hudson et al. 2019). In our workflow, the mollusks were found everywhere in the aquaria and not only on the bottom. Therefore, due to the grazing and feeding behavior, we assess *L. stagnalis* as an important vector for a trophic transfer of wMWCNTs.

### Risk assessment

Selck et al. (2016) explained that many studies concerning nanomaterials were carried out for short-term exposures (24–48 h) with a lack of chronic and delayed effects to organisms. Beyond that, they highlighted the necessity to investigate modified nanomaterials together with long-term experiments for a more comprehensive knowledge about fate, bioaccumulation, and effects of nanomaterials to the environment. Consequently, we developed a workflow as short-term and long-term tests with quantitative and qualitative endpoints to support ecotoxicological risk assessment for weathered MWCNTs.

Regarding environmental risk assessment, the production volume of 100 to 1000 t per year (Piccinno et al. 2012) is a relevant information. According to REACH, chronic tests of substances and products are necessary if the production volume exceeds the threshold of 100 t per year (REACH Annex IX, 9.1.5; William, Berninger and Brooks 2011; Foth and Hayes 2008). Long et al. (2012) and Verneuil et al. (2015) investigated algae acute toxicity using *C. vulgaris* and determined an *EC50*_(96 h)_ of 41 ± 3.0 mg/L and *N. palea* about 118 mg/L after 48 h. For *D. magna*, an *EC50*_(48 h)_ of 14 ± 0.3 mg/L was investigated by Sanchis et al. (2016) due to the immobilization assay (OECD 202 and ISO 6341). Furthermore, a chronic test was performed with *D. magna* over 14 days, and an *EC50* of 4.3 mg/L was determined (Stanley et al. 2015). For *D. rerio*, a lowest observed effect concentration (*LOEC*) of 60 mg/L in embryos was found after 48 and 72 h (Asharani et al. 2008). Hence, *D. magna* is the most sensitive of these species with an effect concentration of 4.3 mg/L. The availability of data comprising acute and one prolonged test results in an uncertainty factor of 100 and an extrapolated *PNEC* of 0.043 mg/L.

Referring to our experiments, the biofilm was affected at a water exposure concentration of 0.1 mg/L, and the corresponding uncertainty factor results in 50; an additional chronic test is available. Thus, a lower *PNEC* of 0.002 mg/L is calculated. Estimating the *MEC* (0.8 ng/L, Maurer-Jones et al. 2013) to *PNEC* ratio, the risk quotient results in 0.0004, i.e., well below 1. Moreover, we can assess that the concentration of 529 mg/144 cm^2^ for benthic biofilm results in a risk quotient of 0.10 by using a *MEC* of 1 mg/kg in sediment (Selck et al. 2016). In our case, no risk was identified for water and sediment exposure, but a careful assumption leads to the conclusion that the risk quotient is getting a thousand times higher in sediment compartments.

### Further research and future insights

Although the primary consumer *L. stagnalis* was significantly influenced after the exposure to wMWCNTs, it fully recovered. Anyhow, *L. stagnalis* indicates a high bioaccumulation potential of the nanotubes and a negative impact on physiological biomarkers and thus stress for the organisms. Further research should focus on the fate of wMWCNTs, whether they remain in the midgut and digestive gland or intracellularly in *L. stagnalis*, and the associated effects.

Van der Zande (2020) described the gut as a barrier, which has received little attention in the past. Consequently, it is temporarily difficult to understand the fate and behavior of wMWCNTs in detail after ingestion. Therefore, more insights about the transmission of wMWCNTs to different tissue parts of the gut should get more attention. Caixeta et al. (2020) refer to significant research gaps concerning, e.g., points of toxicokinetic, which means especially the tissue distribution, detoxification processes, and metabolism of nanomaterials in snails. Our results support these important suggestions. Moreover, Amorim et al. (2019) emphasized also the importance of histological examinations of a hazard identification for risk assessment.

In our study, we did not detect intracellular wMWCNTs or unusual granules in intestinal epithelial enterocytes (data not shown), but they may still be found in the cells of the digestive gland, which is currently under investigation. This will help to answer whether wMWCNTs are taken up intracellularly in granules, as described by Elangovan et al. (2000), and contribute to understand the fate of wMWCNTs in granules and the associated possible sublethal effects in organisms more precisely. In addition, this may also provide insights into the possible barrier function of the gut, such as impaired endocytosis or phagocytosis, trophic membranes, or other yet unknown mechanisms. Finally, as Caixa et al. (2020) have recommended to evolve standard protocols for further research, our workflow is the first step to establish such a standard protocol for further tests with nanomaterials concerning hazard identification.

## Conclusion

Benthic biofilm as food source of *L. stagnalis* is a sink for wMWCNTs which form a dense network agglutinated by extracellular polymeric substances. Thus, wMWCNTs are transported via the food chain to the primary consumer *L. stagnalis* feeding on the biofilm conglomerate. Moreover, after ingestion, the snails incorporate the nanotubes extracellularly in the gut lumen. The level of triglycerides in the snails responded to the exposure to the nanotubes and can serve as stress indicator, whereas the glycogen concentration was not significantly changed. Stress was also indicated by a significant inhibition of growth after 24 days of exposure with 10 mg/L wMWCNTs. Subsequent detailed histological analyses showed that the midgut gland, also referred to as *hepatopancreas*, may be a crucial compartment for the fate of wMWCNTs. The grazing behavior of the snails all over the aquaria renders the quantification of wMWCNT uptake difficult. For this, a quantitative biomagnification study with radiolabeled ^14^C-wMWCNTs and with detailed histological studies of the intestine and digestive gland, in particular the subcellular distribution of wMWCNTs, will be performed and reported separately.

## Data Availability

On inquiry, the data presented in this study is available from the authors.
